# Supplementary material for: Population Genomic Structure of Sorghum Landraces Across Landscape, Environment and Culture
Source: Mol Ecol. 2026 Mar 6;35(5):e70287. doi: 10.1111/mec.70287 (PMC12965196; doi:10.1111/mec.70287)
Supplement: Supplementary file 1 — Figure S1: Scatterplot of travel time and genetic distance 1806 landraces. Figure S2: Scatterplot of travel time in subregions East Africa (R 2 = 0.140), South Africa (R 2 = 0.131), West Africa (R 2 = 0.111) and South Asia (R 2 = 0.078). Figure S3: Variance partitioning for predictor variables moisture, temperature, travel time, language using SNPs as the multivariate response factor. Figure S4: Map of 1806 landraces and associated 42 unique language families. Figure S5: Neighbour‐joining tree depicting genetic relatedness (Hamming distance) among West African landraces. Figure S6: Scatterplot of geographic distance and genetic distance for 11 varieties present among 491 West African landraces without guinea margaritiferum varieties. Figure S7: Scatterplot of geographic distance and genetic distance for four varieties present in West Africa. Figure S8:Scatterplot of geographic distance (km) and travel time (hours) for paired sorghum accessions. Points are stratified by continent. Table S1: Metadata/coordinates for waypoints used in travel time calculations. [file MEC-35-e70287-s001.pdf]

Supplemental Materials for:

"Population genomic structure of sorghum landraces across landscape, environment and culture"

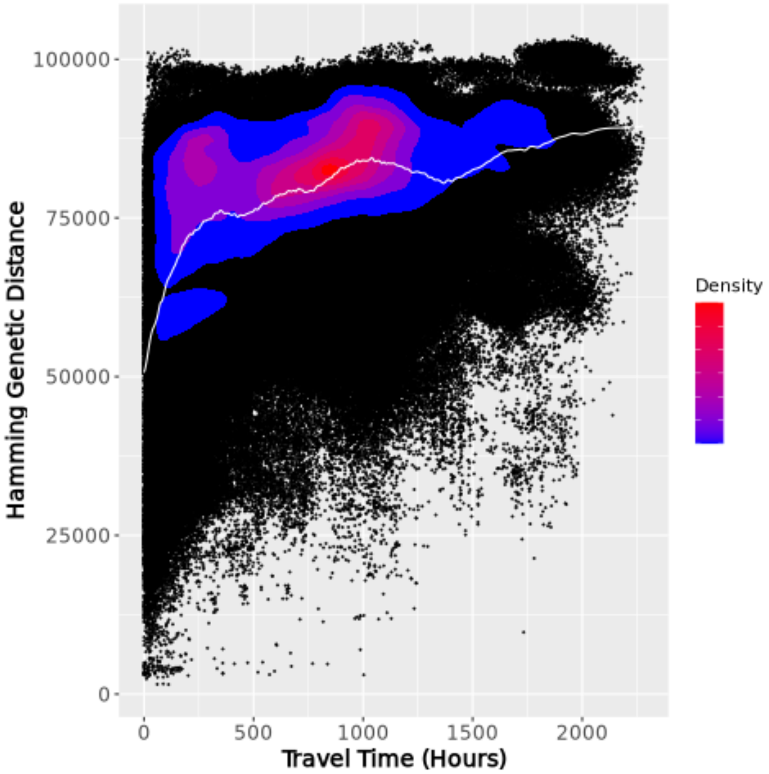

Figure S1. Scatterplot of travel time and genetic distance 1,806 landraces. Spline (white line) added to show distribution pattern. R^2=0.188

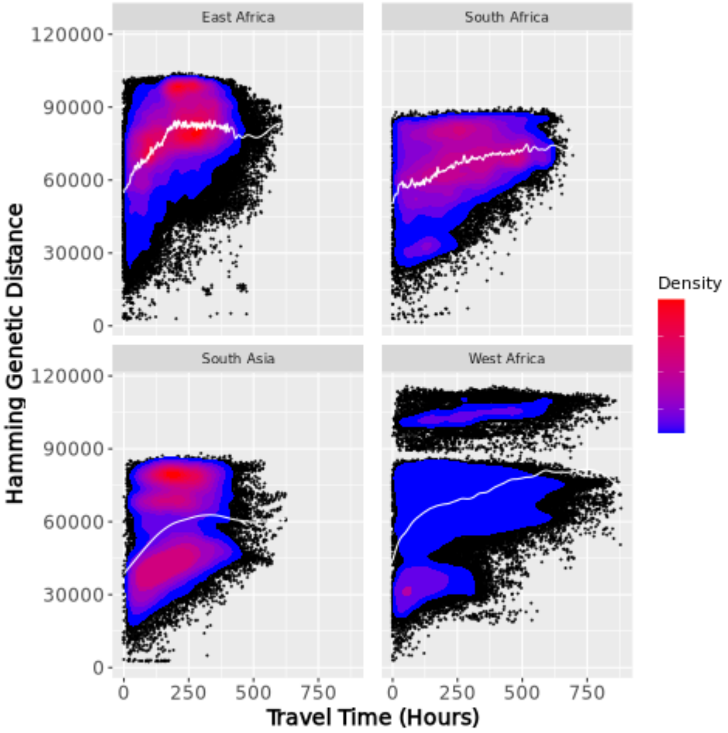

Figure S2. Scatterplot of travel time in subregions East Africa (R^2= 0.140), South Africa (R^2= 0.131), West Africa (R^2= 0.111) and South Asia (R^2= 0.078). Spline (white line) added to show distribution pattern.

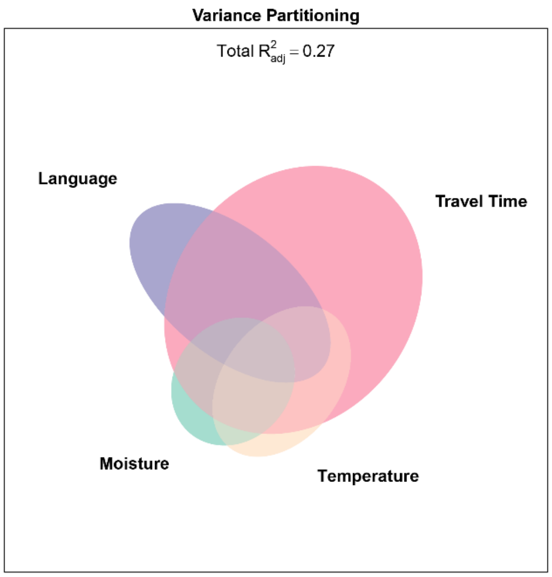

Figure S3. Variance partitioning for predictor variables moisture, temperature, travel time, language using SNPs as the multivariate response factor. Ellipse size corresponds to the proportion of variation explained by variables in each category, with the ellipse overlap indicating collinear variation in predictors that explained variation in SNPs.

R^2_{adj} = 0.27. Residuals = 0.73

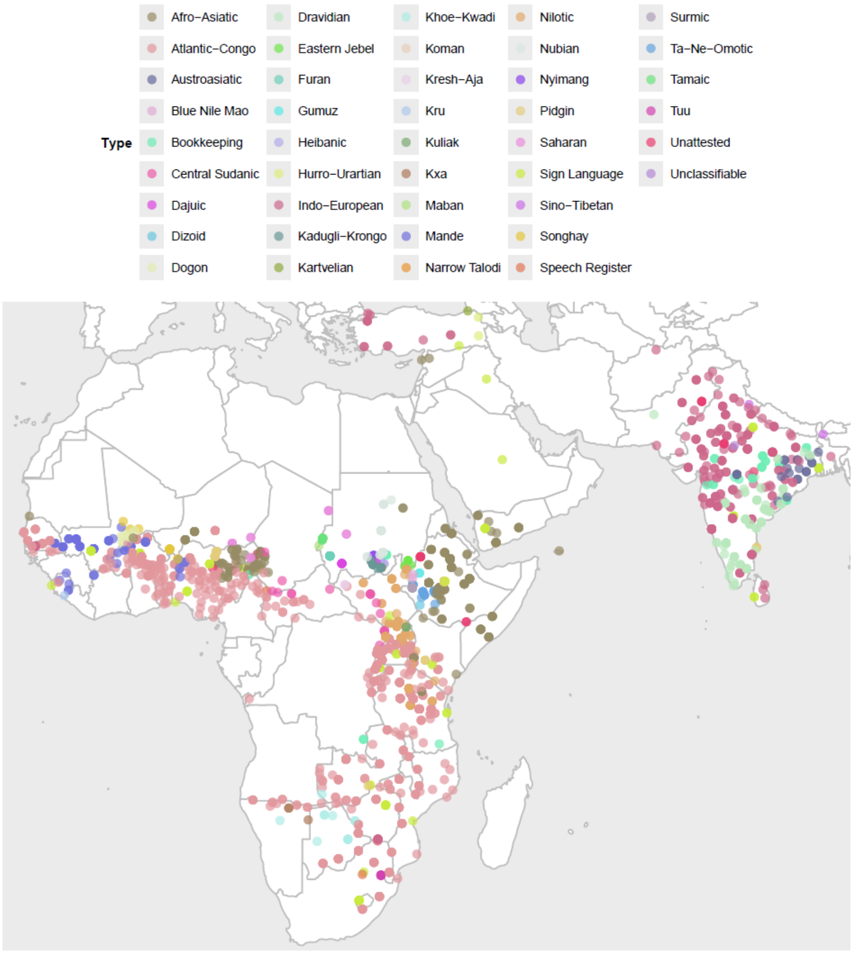

Figure S4. Map of 1806 landraces and associated 42 unique language families.

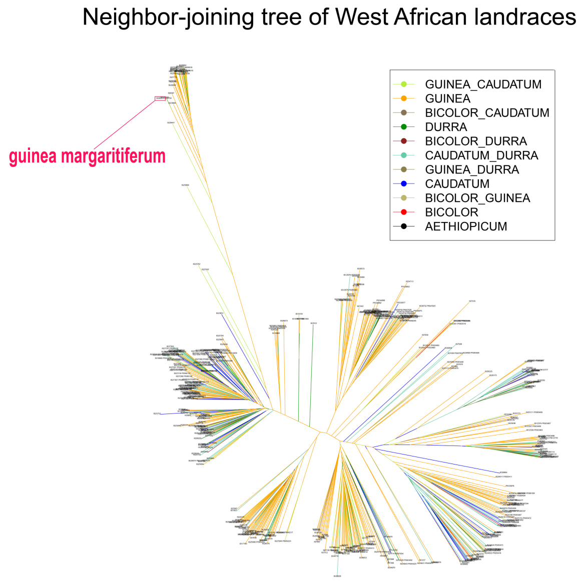

Figure S5. Neighbor-joining tree depicting genetic relatedness (Hamming distance) among West African landraces. Legend colors denote variety names. The landrace "IS3620," which is categorized as the *guinea margaritiferum* subrace, is highlighted.

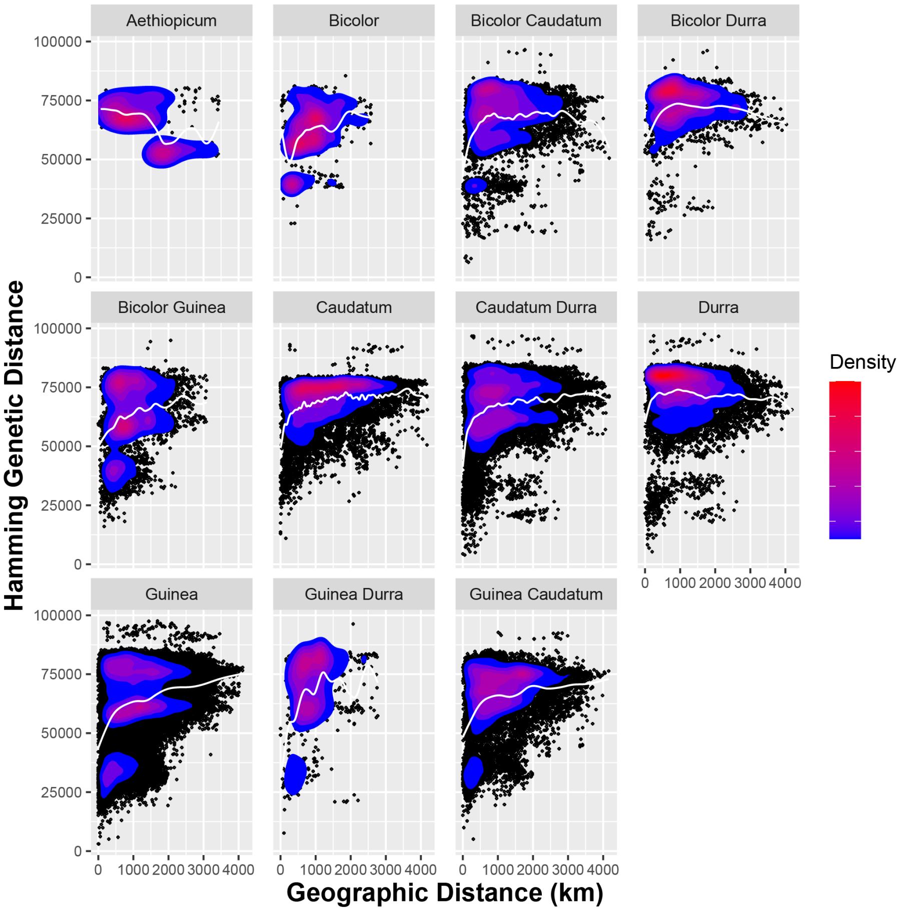

Figure S6. Scatterplot of geographic distance and genetic distance for 11 varieties present among 491 West African landraces without guinea margaritiferum varieties. The 30 margariteferum landraces removed were identified using hclust (k=2) dendogram. Spline (white line) added to show distribution pattern.

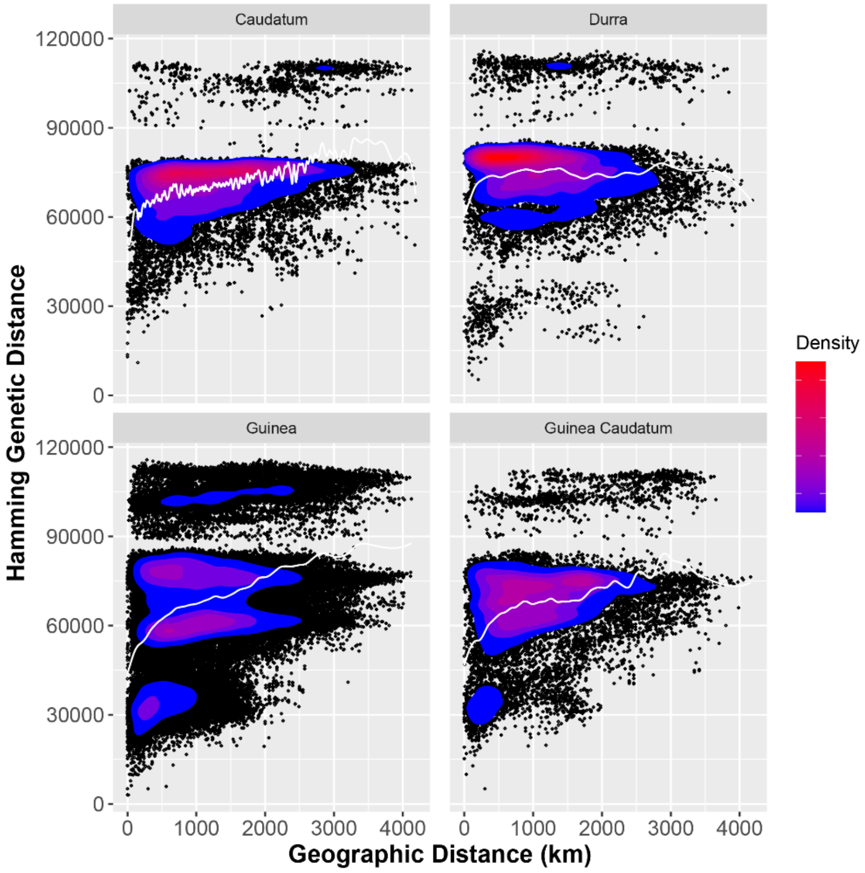

Figure S7. Scatterplot of geographic distance and genetic distance for 4 varieties present in West Africa. The distribution pattern of the Guinea variety reflects the same divergence pattern present in West Africa. Spline (white line) added to show distribution pattern.

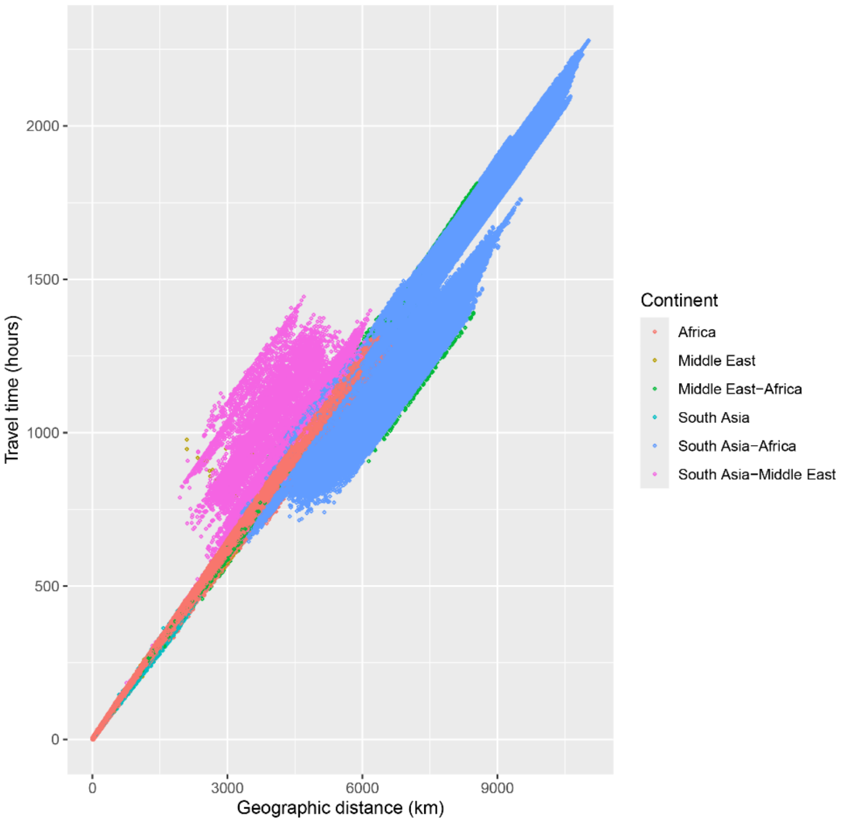

Figure S8. Scatterplot of geographic distance (km) and travel time (hours) for paired sorghum accessions. Points are stratified by continent (within and between continents), as indicated by color in legend.

Table S1. Metadata/Coordinates for waypoints used in travel time calculations.

Waypoint	Country	Longitude	Latitude
Suez	Egypt	32.549494	29.966183
Mombasa_FortJesus	Kenya	39.679722	-4.062778
Saylac_Zeila	Somalia	43.475322	11.351808
KilwaKisiwani	Tanzania	39.512800	-8.960000
SabaeanLane_AlLuhayyah	Yemen	42.700508	15.708842
StoneTown_Zanzibar	Tanzania	39.191300	-6.162400
Mogadishu	Somalia	45.341944	2.039167
